# Supplementary material for: Location decision of low-altitude service station for transfer flight based on modified immune algorithm
Source: PeerJ Comput Sci. 2023 Nov 1;9:e1624. doi: 10.7717/peerj-cs.1624 (PMC10702995; doi:10.7717/peerj-cs.1624)
Supplement: Supplemental Information 1 [file peerj-cs-09-1624-s001.docx]

function lianxudongtai(radius) {

 ***$***.ajax({
 url:'/city/lianxudongtai',
 data:{radius:radius},
 type:'get',
 datatype:"json",
 async:"false",
 success:function(result){
 var str="";
 var tbody = ***document***.getElementById("siteSelection").contentWindow.document.getElementById('airportsInCityBody');
 for (var item in result) {
 if (result[item].count!=0) {
 str += "<tr>" +
 "<td align='center'>" + result[item].city.cityName + "</td>" +
 "<td align='center'>" + result[item].city.cityLon + "</td>" +
 "<td align='center'>" + result[item].city.cityLat + "</td>" +
 "<td align='center'>" + result[item].count + "</td>" +
 "</tr>";
 var entity = new Cesium.Entity({
 id: 'city'+result[item].city.idcity,
 name: result[item].city.cityName,
 show: true,
 position: Cesium.Cartesian3.fromDegrees(result[item].city.cityLon,result[item].city.cityLat),
 point: new Cesium.PointGraphics({
 show: false,
 pixelSize: 5,
 heightReference: Cesium.HeightReference.NONE,
 color: new Cesium.Color(255, 255, 0, 1),
 outlineColor: new Cesium.Color(0, 0, 0, 0),
 outlineWidth: 1,
 scaleByDistance: new Cesium.NearFarScalar(0, 1, 5e10, 1),
 translucencyByDistance: new Cesium.NearFarScalar(0, 1, 5e10, 1),
 distanceDisplayCondition: new Cesium.DistanceDisplayCondition(0, 4.8e10),

 }),
 ellipse: {
 semiMinorAxis: radius,
 semiMajorAxis: radius,
 height: 200,
 material: Cesium.Color.RED,
 outline: true, // height must be set for outline to display
 outlineColor: Cesium.Color.WHITE,
 outlineWidth: 8,
 width: 10,
 heightReference: Cesium.HeightReference.CLAMP_TO_GROUND,
 fill: false
 },

 label: {
 text: result[item].city.cityName,
 font: '16pt monospace',
 color: new Cesium.Color(255, 255, 0, 1),
 style: Cesium.LabelStyle.FILL_AND_OUTLINE,
 outlineWidth: 5,
 verticalOrigin: Cesium.VerticalOrigin.BOTTOM,
 pixelOffset: new Cesium.Cartesian2(0, -9),
 },
 billboard: {
 image: "../static/image/dingwei.png",
 width: 16,
 height: 16,
 },
 });
 ***viewer***.entities.add(entity);

 }
 tbody.innerHTML = str;
 }

}});

}

clear all;

close all;

clc;

%Coordinate data

C = [ 30.67 104.06

30.88 104.32

30.57 104.94

30.2 103.29

30.8 103.86

30.82 104.13

26.9 102.15

26.9 101.56

30.97 103.81

31.04 103.61

30.99 103.94

31.13 104.16

30.99 104.25

30.42 103.78

30.42 103.47

30.58 103.53

30.63 103.69

31.48 104.73

31.8 104.7

32.59 105.21

32.42 104.52

32.44 105.86

32.25 106.33

32.03 105.45

31.64 105.16

31.1 105.06

31.23 105.35

30.9 105.31

30.52 105.58

30.78 105.74

31.06 104.68

31.13 104.37

31.32 104.19

31.64 104.41

31.89 104.44

29.59 105.04

30.3 105.02

30.12 105.3

29.57 104.7

29.81 104.85

30.19 104.6

30.38 104.53

29.64 105.25

29.77 104.56

29.24 104.97

28.87 104.96

28.71 105.06

28.77 105.38

28.96 105.46

28.79 105.78

28.91 105.39

28.03 105.79

28.19 105.44

28.6 104.91

28.36 105.06

28.38 104.81

28.4 104.52

28.16 104.53

28.68 104.15

29.59 103.73

29.75 103.59

29.95 103.38

30.04 103.53

29.86 103.81

30.05 103.81

30.22 103.83

29.67 104.06

30 104.09

29.21 103.93

28.96 103.98

29.62 103.5

28.87 103.53

29.23 103.25

29.24 103.13

29.7 107.36

30.36 107.34

29.89 107.7

29.98 108.13

28.47 108.97

28.85 108.75

29.53 108.81

29.29 108.19

29.29 108.72

29.15 107.13

30.83 108.35

31.23 108.39

31.98 108.67

31.42 109.6

31.1 109.86

31.06 109.52

30.99 108.89

30.33 108.03

30.66 107.78

30.8 106.06

31.75 105.96

31.75 105.97

31.52 106.38

31.34 106.03

31.01 105.84

31.07 106.57

31.04 106.44

30.48 106.61

30.55 106.43

30.38 106.3

30.41 106.74

31.23 107.49

32.07 108.06

31.39 107.71

31.1 107.87

30.36 106.91

30.75 107.21

30.85 106.94

32.36 106.83

31.86 106.73

31.59 107.11

31.95 108.24

32 108.18

29.97 102.97

30.17 102.91

30.09 103.06

29.79 102.81

29.4 102.66

29.21 102.38

30.09 102.78

30.36 102.84

31.92 102.22

31.79 102.55

31.93 101.72

33.62 102.94

32.06 102.95

32.64 103.61

33.23 104.19

31.46 103.61

31.42 103.16

30.97 102.34

31.48 102.03

32.3 100.97

31.67 103.89

30.04 101.95

31.38 100.65

31.64 99.96

30.96 100.28

32.23 98.83

31.81 98.57

33.01 98.06

32.3 100.35

29.92 102.25

30.85 101.87

29.01 101.53

30.03 101

30.99 101.14

30.03 100.28

28.93 99.78

29.04 100.31

30 99

28.71 99.25

27.92 102.29

28.03 102.83

28.96 102.74

28.21 103.62

27.07 102.76

26.74 102.55

26.67 102.21

27.4 102.15

28.33 103.14

27.73 103.22

27.7 102.8

27.38 102.52

28.33 102.42

28.66 102.49

27.42 101.51

28.58 102.15

27.9 101.25

29.35 104.77]

N=size(C,1); %Number of cities

D=zeros(N); %Any two city distance interval matrix

%Find the distance matrix of any two cities

for i=1:N

for j=1:N

D(i,j)=((C(i,1)-C(j,1))^2+(C(i,2)-C(j,2))^2)^0.5;

end

end

NP=200; %Number of immune individuals

G=1000; %Maximal immune algebra

f=zeros(N,NP); %For stock

for i=1:NP

f(:,i)=randperm(N); %Generate initial population randomly

end

len=zeros(NP,1); %Storage path length

for i=1:NP

len(:,i)=func3(D,f(:,i),N); %Calculated path length

end

[Sortflen,Index]=sort(len);

Sortf=f(:,Index); %Population individual ranking

gen=0; %Immunological algebra

Nc1=10; %Clone count

%Immune circulation

while gen<G

for i=1:NP/2

a=Sortf(:,i);

Ca=repmat(a,1,Nc1);

for j=1:Nc1

p1=floor(1+N*rand());

p2=floor(1+N*rand());

while p1==p2

p1=floor(1+N*rand());

p2=floor(1+N*rand());

end

tmp=Ca(p1,j);

Ca(p1,j)=Ca(p2,j);

Ca(p2,j)=tmp;

end

Ca(:,1)=Sortf(:,i); %Keep the clone source individual

%Clonal inhibition, retention of the highest affinity individuals

for j=1:Nc1

Calen(j)=func3(D,Ca(:,j),N);

end

[SortCalen,Index]=sort(Calen);

SortCa=Ca(:,Index);

af(:,i)=SortCa(:,1);

alen(i)=SortCalen(1);

end

%Population refresh

for i=1:NP/2

bf(:,i)=randperm(N);

blen(i)=func3(D,bf(:,i),N);

end

%The immune population merges with the new population

f=[af,bf];

len=[alen,blen];

[Sortlen,Index]=sort(len);

Sortf=f(:,Index);

gen=gen+1;

trace(gen)=Sortlen(1);

end

%Output optimization result

Bestf=Sortf(:,1);

Bestlen=trace(end);

figure

for i=1:(N-1)

plot([C(Bestf(i),1),C(Bestf(i+1),1)],...

[C(Bestf(i),2),C(Bestf(i+1),2)],'bo');

hold on;

end

plot([C(3,1),C(128,1),C(170,1)],[C(3,2),C(128,2),C(170,2)],'ro');

title(['best：',num2str(trace(end))]);

figure

plot(trace)

xlabel('iterations')

ylabel('targt')

title('Affinity evolutionary curve')

%Calculate the total length of the route

function len=func3(D,f,N)

len=D(f(N),f(1));

for i=1:(N-1)

len=len+D(f(i),f(i+1));

end

end

package com.kg.fss.controller;

/**

* @program: IAArg

* @description: test

* @author: plf

* @created: 2023/06/20 15:56

*/

import com.kg.fss.entity.City;

import com.kg.fss.util.Algorithm;

import java.io.BufferedReader;

import java.io.FileReader;

import java.util.Arrays;

import static java.lang.Double.POSITIVE_INFINITY;

public class siteSelection {

public static void main(String[] args) throws Exception {

/*

* Read data

*/

BufferedReader br = new BufferedReader(new FileReader("C:\\javaProject\\fss\\src\\main\\java\\com\\kg\\fss\\util\\input_assgin01_03.dat"));

String s = null;

int size = Integer.parseInt(br.readLine());

/*

* 1 Define the candidate point coordinates and the weights corresponding to each point

*/

double[] x = new double[size];

double[] y = new double[size];

double[] xweight = new double[size];

double[] yweight = new double[size];

String[] city = new String[size];

int i = 0;

while((s = br.readLine()) != null){

String[] a = s.split(",");

city[i]= a[3];

x[i] = Double.parseDouble(a[0]);

y[i] = Double.parseDouble(a[1]);

xweight[i] = Double.parseDouble(a[2]);

yweight[i] = Double.parseDouble(a[2]);

i++;

}

siteSelection siteSelection = new siteSelection();

double[] xtemp = new double[size];

xtemp= Arrays.copyOf(x,size);

double[] ytemp = new double[size];

ytemp=Arrays.copyOf(y,size);

City resultCity = siteSelection.initarea(x, y, xweight, yweight,xtemp,ytemp,city);

System.out.println("The result is:");

System.out.println(resultCity.getCityLat());

System.out.println(resultCity.getCityLon());

System.out.println(resultCity.getCityName());

}

/**

* Quick sort (at the same time, the weights corresponding to each point are adjusted in turn)

*

* @param attr

* @param weight

* @param low

* @param height

*/

public void pxres(double[] attr, double[] weight, int low, int height){

double temp = 0;

double temp1 =0;

int i = low;

int j = height;

if(low < height){

temp = attr[low];

temp1 = weight[low];

while(i != j){

while(j > i && attr[j] >= temp){

--j;

}

if(i < j){

attr[i] = attr[j];

weight[i] = weight[j];

++i;

}

while(i < j && attr[i] < temp){

++i;

}

if(i < j){

attr[j] = attr[i];

weight[j] = weight[i];

--j;

}

}

attr[i] = temp;

weight[i] = temp1;

pxres(attr, weight, low, i - 1);

pxres(attr, weight, i + 1, height);

}

}

/**

* Find the coordinates corresponding to the weighted median for each axis

* @param addr

* @param Weights

* @param zhou

* @return

*/

public double axis(double [] addr, double [] Weights, String zhou){

/*

* Quickly sort each axis coordinate and adjust the corresponding weights

*/

pxres(addr, Weights, 0, addr.length - 1);

/*

* 3 Sum of weights of all candidate points

*/

double sumweight = 0;

for(int i = 0; i < Weights.length; i++){

sumweight += Weights[i];

}

/*

* 4 Find the weighted median in the latitude direction

*/

double sum = 0;

for(int i = 0; i < Weights.length; i++){

sum += Weights[i];

if(sum >= sumweight / 2){

return addr[i];

}

}

return 0;

}

/**

* Method of finding flight service station coordinates

* @param Xaxis

* @param Yaxis

* @param XWeights

* @param YWeights

*/

public City initarea(double[] Xaxis, double[] Yaxis, double[] XWeights, double[] YWeights,double[] x,double[] y,String[] citys){

/*

* 2 Latitude and longitude are processed separately (Note: the weights corresponding to the order of the corresponding axes are adjusted accordingly to keep them one-to-one)

* 2.1 Quick sort of latitude coordinate points

*/

double px = axis(Xaxis, XWeights, "Latitude");

/*

* 2.2 Quick sort of longitude coordinate points

*/

double py = axis(Yaxis, YWeights, "longitude");

/*

2.3Determine the location of the nearest town point

*/

City result = new City();

result = searchCity(px,py,x,y,citys);

/*

* Print the service station location

*/

return result;

}

/**

*

* @param x

* @param y

* @param xs

* @param ys

* @return

*/

public City searchCity(double x, double y, double[] xs,double[] ys,String[] citys){

City result = new City();

double minDistance = POSITIVE_INFINITY;

for (int i=0;i<xs.length;i++) {

double tempDistance = Algorithm.GetDistance(x,y,xs[i],ys[i]);

if (tempDistance<minDistance){

minDistance=tempDistance;

result.setCityLat(xs[i]);

result.setCityLon(ys[i]);

result.setCityName(citys[i]);

}

}

return result;

}

}
